# The dipeptidyl peptidase-4 inhibitor, linagliptin, improves cognitive impairment in streptozotocin-induced diabetic mice by inhibiting oxidative stress and microglial activation

**DOI:** 10.1371/journal.pone.0228750

**Published:** 2020-02-07

**Authors:** Makoto Ide, Noriyuki Sonoda, Tomoaki Inoue, Shinichiro Kimura, Yohei Minami, Hiroaki Makimura, Eiichi Hayashida, Fuminori Hyodo, Mayumi Yamato, Ryoichi Takayanagi, Toyoshi Inoguchi

**Affiliations:** 1 Department of Internal Medicine and Bioregulatory Science, Graduate School of Medical Sciences, Kyushu University, Fukuoka, Japan; 2 Innovation Center for Medical Redox Navigation, Kyushu University, Fukuoka, Japan; 3 Fukuoka City Health Promotion Support Center, Fukuoka, Japan; Max Delbruck Centrum fur Molekulare Medizin Berlin Buch, GERMANY

## Abstract

**Objective:**

Accumulating epidemiological studies have demonstrated that diabetes is an important risk factor for dementia. However, the underlying pathological and molecular mechanisms, and effective treatment, have not been fully elucidated. Herein, we investigated the effect of the dipeptidyl peptidase-4 (DPP-4) inhibitor, linagliptin, on diabetes-related cognitive impairment.

**Method:**

Streptozotocin (STZ)-induced diabetic mice were treated with linagliptin (3 mg/kg/24 h) for 17 weeks. The radial arm water maze test was performed, followed by evaluation of oxidative stress using DNP-MRI and the expression of NAD(P)H oxidase components and proinflammatory cytokines and of microglial activity.

**Results:**

Administration of linagliptin did not affect the plasma glucose and body weight of diabetic mice; however, it improved cognitive impairment. Additionally, linagliptin reduced oxidative stress and the mRNA expression of NAD(P)H oxidase component and *TNF-α*, and the number and body area of microglia, all of which were significantly increased in diabetic mice.

**Conclusions:**

Linagliptin may have a beneficial effect on diabetes-related dementia by inhibiting oxidative stress and microglial activation, independently of glucose-lowering.

## Introduction

The number of people with dementia is increasing with the aging of the world’s population. It has been reported that diabetes is an independent risk factor for cognitive impairment[1,2], and dementia has been recognised as a complication of diabetes[3]. However, its detailed pathogenesis remains unknown. Thus, it is important to further examine the mechanisms underlying diabetes-related dementia and explore effective treatment strategies. Hyperglycaemia causes oxidative stress, which plays a pivotal role in the development of diabetes complications[4]. Although microglia, immune cells in the brain, are neuroprotective under normal conditions, they produce proinflammatory cytokines and reactive oxygen species (ROS) when activated by inflammation, nerve damage or infection[5]. We have recently reported that diabetes-related cognitive impairment is at least partially caused by oxidative stress via microglial activation in the brain[6].

Dipeptidyl peptidase-4 (DPP-4) inhibitors are a class of drugs approved for the treatment of type 2 diabetes[7]. DPP-4 inhibitors upregulate the incretin hormone, glucagon-like peptide-1 (GLP-1), and gastric inhibitory polypeptide (GIP), which stimulate insulin secretion in response to increased blood glucose levels[8,9]. Although previous studies have reported that DPP-4 inhibitors improve cognitive dysfunction[10,11], the mechanism has not been fully revealed.

In this study, we showed that the DPP-4 inhibitor, linagliptin, improved cognitive impairment, reduced oxidative stress, and suppressed microglial activation in streptozotocin (STZ)-induced diabetic mice, which could evaluate the effect of DPP-4 inhibitor independently of glucose-lowering.

## Materials and methods

The methods used in this paper overlap those used in a previously published article[6].

### Animals

Male C57BL/6J mice were purchased from Charles River (Yokohama, Japan) and were bred under pathogen-free conditions at the Center of Biomedical Research, Research Center for Human Disease Modeling, Graduate School of Medical Sciences, Kyushu University (Fukuoka, Japan). All protocols were reviewed and approved by the Committee on the Ethics of Animal Experiments, Graduate School of Medical Science, Kyushu University. All methods were performed in accordance with the relevant guidelines and regulations. All efforts were made to minimise the number of animals used and their suffering. Diabetes was induced in 7-week-old mice by administering a single intraperitoneal injection of STZ (Sigma-Aldrich, St. Louis, MO, USA) at a dose of 100 mg/kg in 0.1 M citrate buffer (CIT), pH 4.5. Mice given a CIT injection alone served as non-diabetic controls. Two weeks after the injection, diabetes was confirmed by the occurrence of hyperglycaemia (> 250 mg/dL blood glucose). At 9 weeks of age, half of the non-diabetic mice (n = 8) and half of the diabetic mice (n = 8) were randomly chosen to receive a powdered form diet supplemented with powdered form linagliptin (3 mg/kg/24 h) for 17 weeks, while the remaining mice consumed a powdered form diet that did not contain linagliptin, for the same duration. In the present study, we used powdered form diet to mix linagliptin. A previous study[12] has shown that linagliptin (3 mg/kg/24 h) caused an approximately 1.5-fold increase in serum active GLP-1 concentration, compared with controls. At the end of the treatment, all 26-week-old mice were anesthetized with isoflurane and sacrificed. Linagliptin was gifted by Boehringer Ingelheim (Ingelheim, Germany).

### Radial arm water maze (RAWM)

In 26-week-old mice after administration of linagliptin for 17 weeks, learning and memory were assessed using the RAWM. For the radial maze test we used a previously described protocol and apparatus[6,13]. The radial-arm water maze consisted of a circular pool measuring 1 m in diameter with six arms 19 cm wide that radiated out from an open central area, with a submerged escape platform located at the end of one of the arms. Spatial cues including a light were present on the wall of the testing room. The escape platform was placed in a different arm each day, forcing the mouse to use working memory to solve the task. In each trial, the mouse started in one arm and allowed to swim for up to 1 min until reaching the platform; the number of errors until the mouse reached the platform was recorded. The mouse was allowed to stay on the platform for 30 s. After the fourth trial the mouse was placed in a cage for 30 min, and then returned to the maze to start the fifth trial to assess memory retention. After three consecutive days of training, the error score was determined as the score in the fifth trial averaged over the next 2 days of testing.

### *In vivo* dynamic nuclear polarization (DNP)-MRI

In vivo redox imaging was performed with a custom in vivo DNP-MRI system, constructed using the external magnet of a commercial EPR spectrometer (JES-ES20, JEOL Ltd.). The external magnetic field B_0_ for EPR irradiation and MRI was fixed at 20 mT, and the radiofrequency of the EPR irradiation and MRI were 527.5 MHz and 793 kHz, respectively. A surface coil (diameter: 20 mm) for EPR irradiation was made for head imaging in this study. Brain oxidative stress was measured by DNP-MRI in 26-week-old mice after administration of linagliptin for 17 weeks. During the procedure, the body temperature of the mice was kept at 37 ± 1 °C with a heating pad. Animals were anaesthetised with isoflurane (4% for induction, 1–2% for maintenance) mixed with medical air (flow rate; 750 mL/min), which flowed into a nose cone fitted to the head. After the anaesthesia, methoxycarbonyl-PROXYL (MCP) was injected into the tail vein at a dose of 1.3 mmol/kg body weight. Immediately after the MCP administration, kinetic data were obtained. Pharmacokinetic DNP-MRI images were obtained at 2, 4, 7, 10, 13 min after injection. Normal MRI images were obtained without EPR irradiation. The DNP-MRI signal change of the whole brain was used for calculating the decay rate. The protocol of this measurement has been described previously[14]. The scanning conditions for the *in vivo* DNP-MRI experiment were as follows: power of EPR irradiation, 9 W; flip angle, 90°; repetition time (T_R_) × echo time (T_E_) × EPR irradiation time (T_EPR_), 500 × 40 × 250 ms; number of averages, 1; slice thickness, 20 mm, phase-encoding steps, 32; field of view (FOV), 40 × 40 mm; and matrix size, 64 × 64 after reconstruction.

### Brain lipid peroxidation

The brain levels of lipid peroxidation were estimated in whole mouse brain homogenates as malondialdehyde (MDA) concentration using the Thiobarbituric acid reactive substances (TBARS) assay kit (JaICA, Shizuoka, Japan) according to the manufacturer’s instructions.

### Tissue processing

Tissue processing was performed according to a previous study[6,15]. The animals were anaesthetised with a mixture of isoflurane (4% for induction, 1–2% for maintenance) and medical air (flow rate; 750 mL/min), which flowed into a nose cone fitted to the animal’s head. They were then perfused transcardially with phosphate-buffered saline (PBS, pH 7.4) followed by a fixative: a mixture of 4% paraformaldehyde (PFA) and 0.1% glutaraldehyde in 0.1 M phosphate buffer for immunostaining. The brains were left *in situ* for 3 h at room temperature, and then removed from the skull. The brains were fixed by immersion in 4% PFA overnight at 4°C, and then immersed in 20% sucrose (pH 7.4) for 24 h at 4°C. Then, 50-μm-thick sections were cut by a vibrating microtome (CM1950; Leica Microsystems, Wetzlar, Germany). To avoid deformation of the sections, they were processed free-floating with extreme caution.

### Immunofluorescence procedure

Immunofluorescence was performed as previously described[6,15]. The cerebral cortex sections were incubated with 1.0% bovine serum albumin in PBS containing 0.3% Triton-X 100 and 0.05% sodium azide for 30 min at room temperature. Then, they were incubated for 3 days at room temperature with rabbit polyclonal anti-ionised calcium binding adaptor protein 1 (Iba1) antibody (1:10,000; Wako, Pure Chemical industries, Osaka, Japan). They were then incubated with fluorescein isothiocyanate (FITC)-conjugated donkey anti-goat IgG antibodies (1:300; Jackson ImmunoResearch Laboratories) for 12 h at 4°C in a dark chamber. Next, the sections were counterstained with Hoechst 33258 (Invitrogen, Carlsbad, CA, USA) in PBS for 15 min in a dark chamber. After washing with PBS, the sections were mounted in Vectashield (Vector laboratories, Peterborough, UK) and examined.

### Cell counting and cell body area analysis of Iba1-positive cells

Twenty Z-stack images were acquired at a thickness of 40 μm separated by 2-μm intervals and converted to one Z-projection image. The images for cell counting and cell body area measurements of Iba1-positive cells were examined using a fluorescence microscope (model BZ-9000, Keyence, Osaka, Japan). We counted the Hoechst 33258-stained nuclei of Iba1-positive microglia using the Cell Counter plugin of ImageJ 1.44 (NIMH; Bethesda, MD, USA). The body area of Iba1-positive cells was examined using a fluorescence microscope (model BZ-9000, Keyence, Osaka, Japan). The average cell number and body area of four images were used as one data.

### RNA extraction and quantitative RT-PCR

Total RNA was isolated from whole brain using SV Total RNA Isolation System (Promega, Madison, WI, USA) following the instructions provided with the kit. The mRNA was amplified by quantitative RT-PCR using GoTaq Master Mix (Promega). The mRNA levels were quantified by real-time RT-PCR using a Roche Light Cycler 480 iCycler system (Roche Diagnostics, Tokyo, Japan). The mRNA expression levels of each gene were normalised to the expression level of the housekeeping gene, *β-actin*. The specific primers for the target genes and housekeeping gene are shown in Supplemental Experimental procedures (S1 Table).

### Statistical analysis

Statistical analysis was performed with Student’s *t*-test or one-way analysis of variance (ANOVA) with Fisher’s protected least significant difference (PLSD) test. *P* ≤ 0.05 was considered statistically significant. Results are presented as mean ± SEM.

## Results

### Linagliptin improves cognitive impairment in diabetic mice

**Fig 1:**
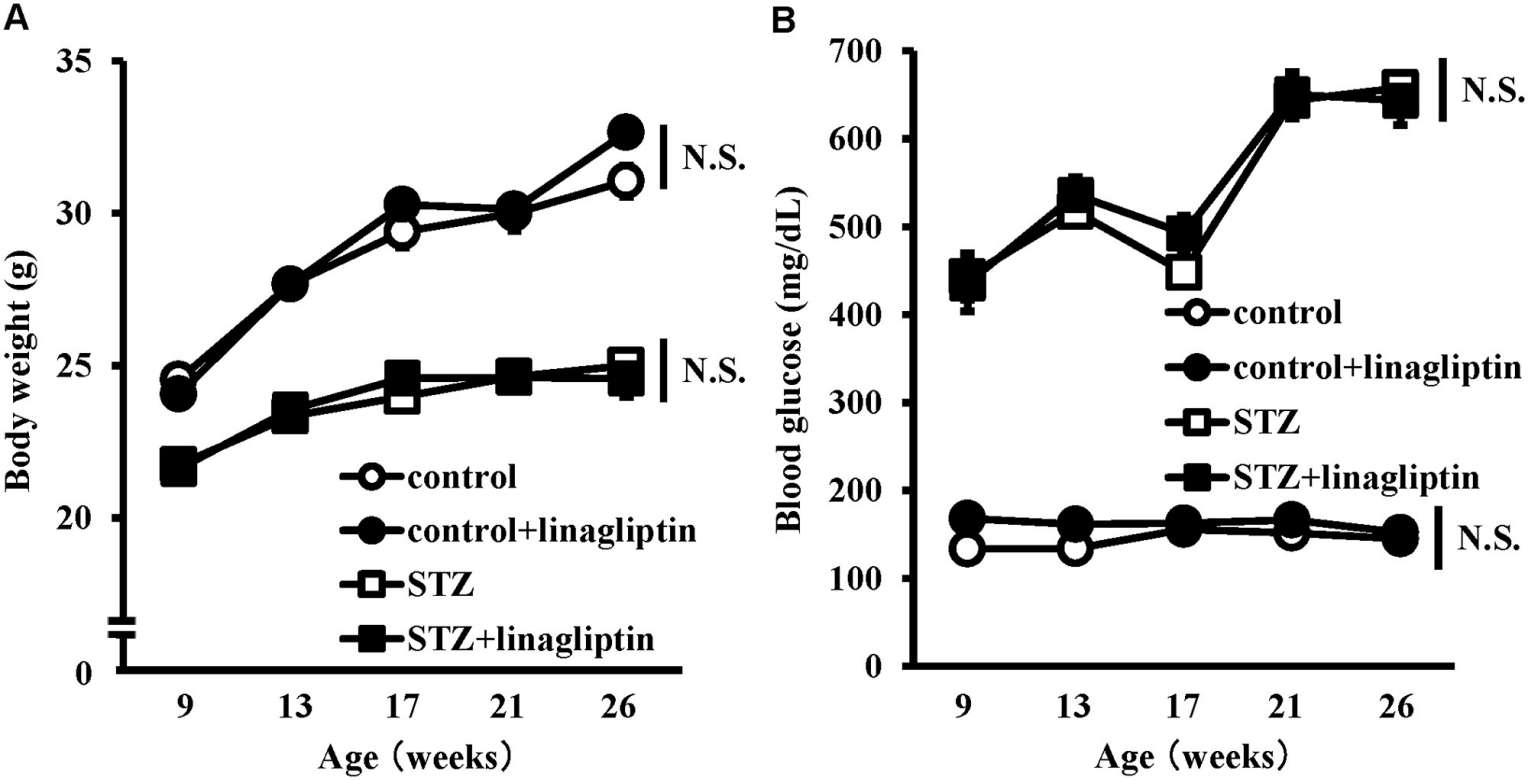
Body weight and blood glucose. Changes in body weight (A) and blood glucose (B) between 9 and 26 weeks of age in control mice (white circles), linagliptin-treated control mice (black circles), diabetic mice (white boxes) and linagliptin-treated diabetic mice (black boxes). Results are expressed as the mean ± SEM (n = 8); N.S., not significant.

**Fig 2:**
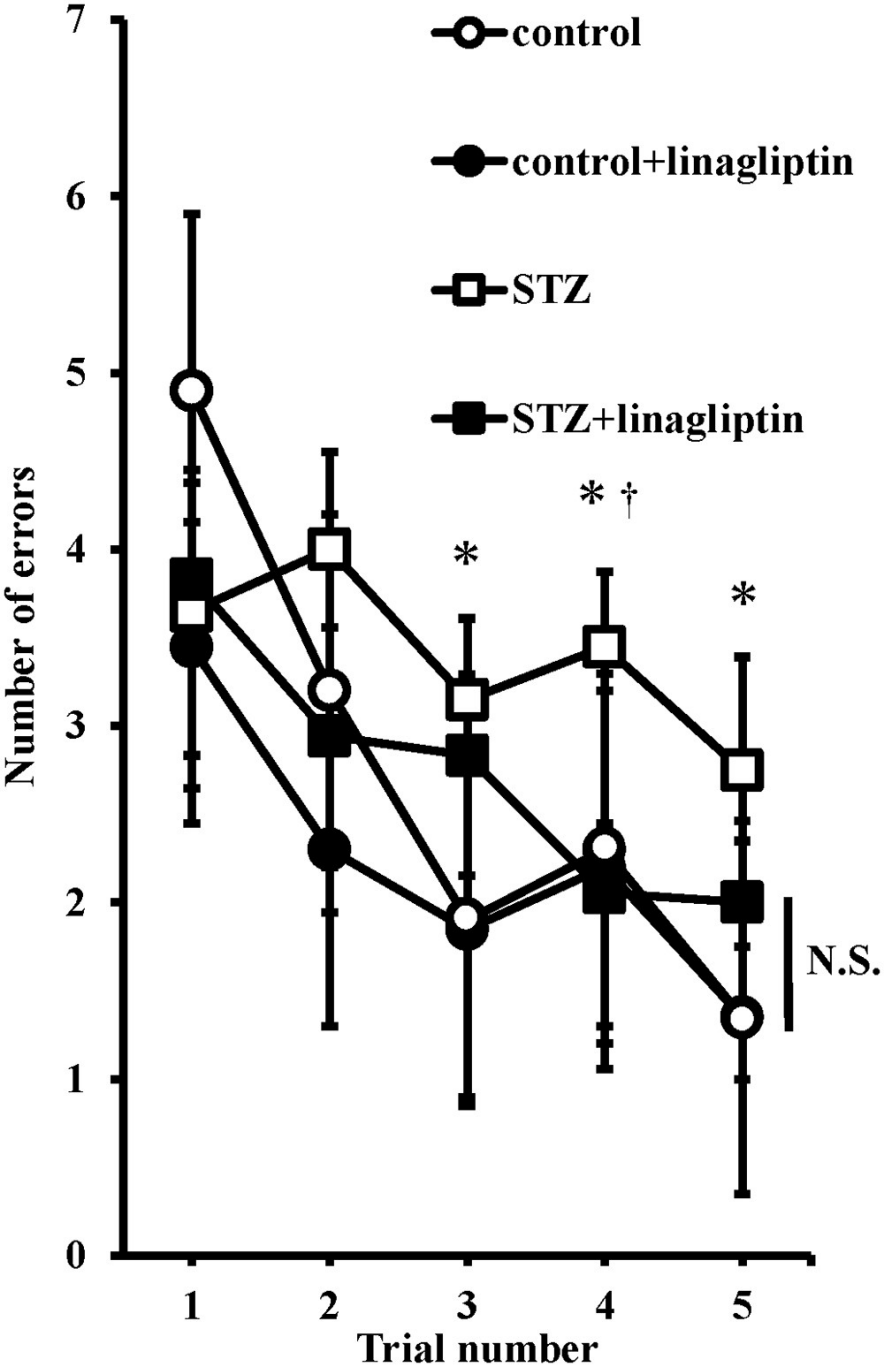
The effect of linagliptin on cognitive impairment. The mean number of errors in the radial arm water maze (RAWM). Four consecutive acquisition trials (trials 1–4) were followed after 30 min by a retention trial (trial 5). Bars represent means ± SEM (n = 8); *P < 0.05 vs. control, †*P* < 0.05 vs. linagliptin-treated diabetic mice (ANOVA). N.S., not significant.

In STZ-induced diabetic mice, the body weight was significantly decreased, and the blood glucose level was significantly higher compared with the control mice (Fig 1). Linagliptin did not affect the body weight and the blood glucose level (Fig 1). To investigate the effects of linagliptin on learning and memory, we tested their performance in the RAWM. RAWM combines elements of a radial-arm maze and a Morris water maze, taking advantage of simple motivation provided by immersion into water together with the benefits of scoring errors. In STZ-induced diabetic mice, the mean number of errors in the acquisition trials (trials 3 and 4) and retention trial (trial 5) was higher compared with the control mice, but there was no difference between the control mice and the linagliptin-treated STZ-induced diabetic mice (Fig 2). In the linagliptin-treated STZ-induced diabetic mice, the mean number of errors in the acquisition trial (trial 4) was lower compared with the STZ-induced diabetic mice (Fig 2). These data indicated that the STZ-induced diabetic mice developed impaired working memory and learning, and linagliptin improved these cognitive impairments.

### Effects of linagliptin treatment on oxidative stress

**Fig 3:**
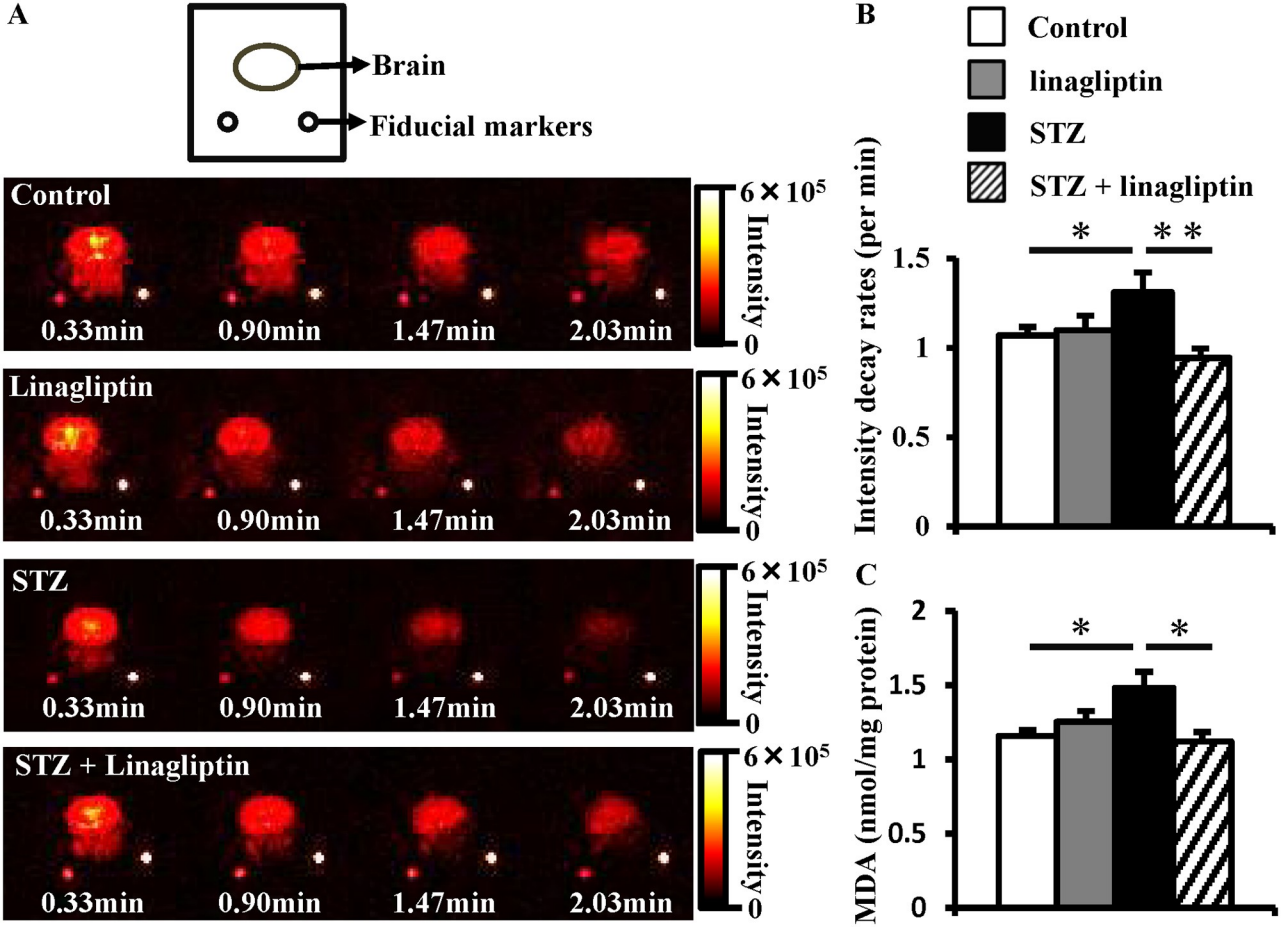
Effect of linagliptin on oxidative stress in the brain. (A) Time-dependent DNP-MRI images of methoxycarbonyl-PROXYL in the head region. (B) The signal intensity decay rates of methoxycarbonyl-PROXYL in the brain. (C) Malondialdehyde (MDA) levels measured using the thiobarbituric acid-reactive substances (TBARS) assay in whole brain homogenates. Bars represent means ± SEM. Control mice (n = 5); linagliptin-treated control mice (n = 4); diabetic mice (n = 6); linagliptin-treated diabetic mice (n = 8). *P < 0.05, **P < 0.01.

We and others have reported that oxidative stress in the brain is associated with diabetes-related cognitive impairment[6,16]. We recently reported that dynamic nuclear polarization-magnetic resonance imaging (DNP-MRI) was useful for evaluating diabetic complication[17]. Thus, we evaluated oxidative stress in the brain of diabetic mice using *in vivo* DNP-MRI. DNP-MRI is a newly developed non-invasive technique for imaging the redox status in living animals by the Overhauser effect[18]. DNP-MRI visualises the tissue redox status by quantifying the intensity of free radicals reacted with nitroxyl radicals. Because the nitroxyl radical, MCP, is a redox sensitive contrast agent in brain imaging[14,19,20], we chose it as the redox-sensitive contrast agent for our study. As the administered MCP reacts with *in vivo*-produced ROS and is consumed, the oxidative stress is accelerated, MCP is consumed quickly and the decay rate of the image intensity increases. In the STZ-induced diabetic mice’s brain, the decay rate was significantly higher than that in the control mice, indicating that oxidative stress in the diabetic brains was elevated. However, in the linagliptin-treated diabetic mice, the decay rate was lower, indicating that the enhanced oxidative stress in the diabetic brains was suppressed by linagliptin administration (Fig 3A and 3B). Additionally, we evaluated brain MDA levels, a naturally occurring product of lipid peroxidation and representative indicator of oxidative stress[21]. MDA levels were markedly increased in the STZ-induced diabetic mice, which was reduced by linagliptin (Fig 3C).

### Effect of linagliptin on microglial activity and mRNA expression of NAD(P)H oxidase components and proinflammatory cytokines

**Fig 4:**
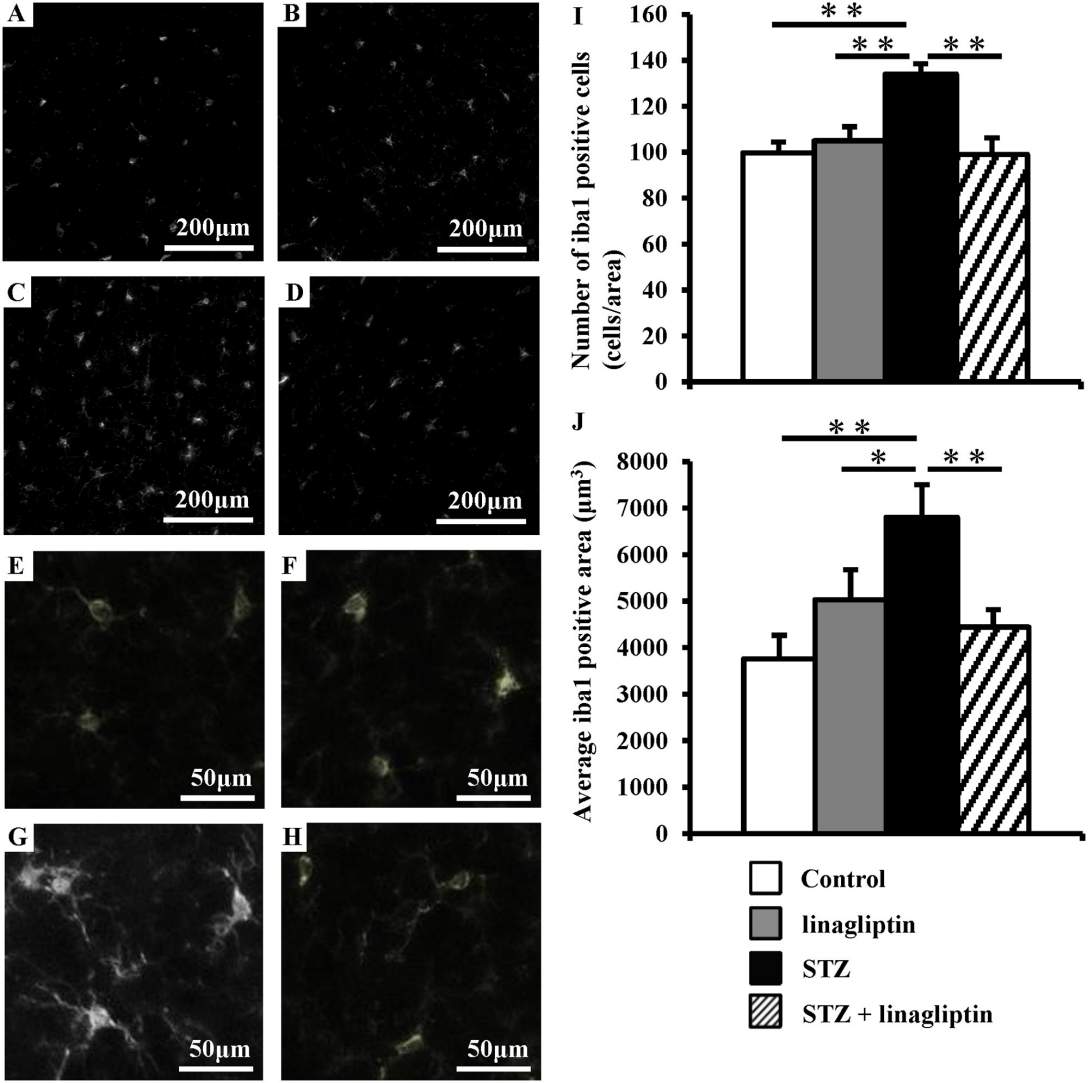
Effect of linagliptin on brain microglia in diabetic mice. Cerebral cortex sections were immunostained with anti-Iba1 antibodies. (A, E) Control mice, (B, F) linagliptin-treated control mice, (C, G) diabetic mice and (D, H) linagliptin-treated diabetic mice. The cell number (I) and body area (J) of Iba1-positive cells in the brains. Bars represent means ± SEM (n = 8). *P < 0.05, **P < 0.01.

**Fig 5:**
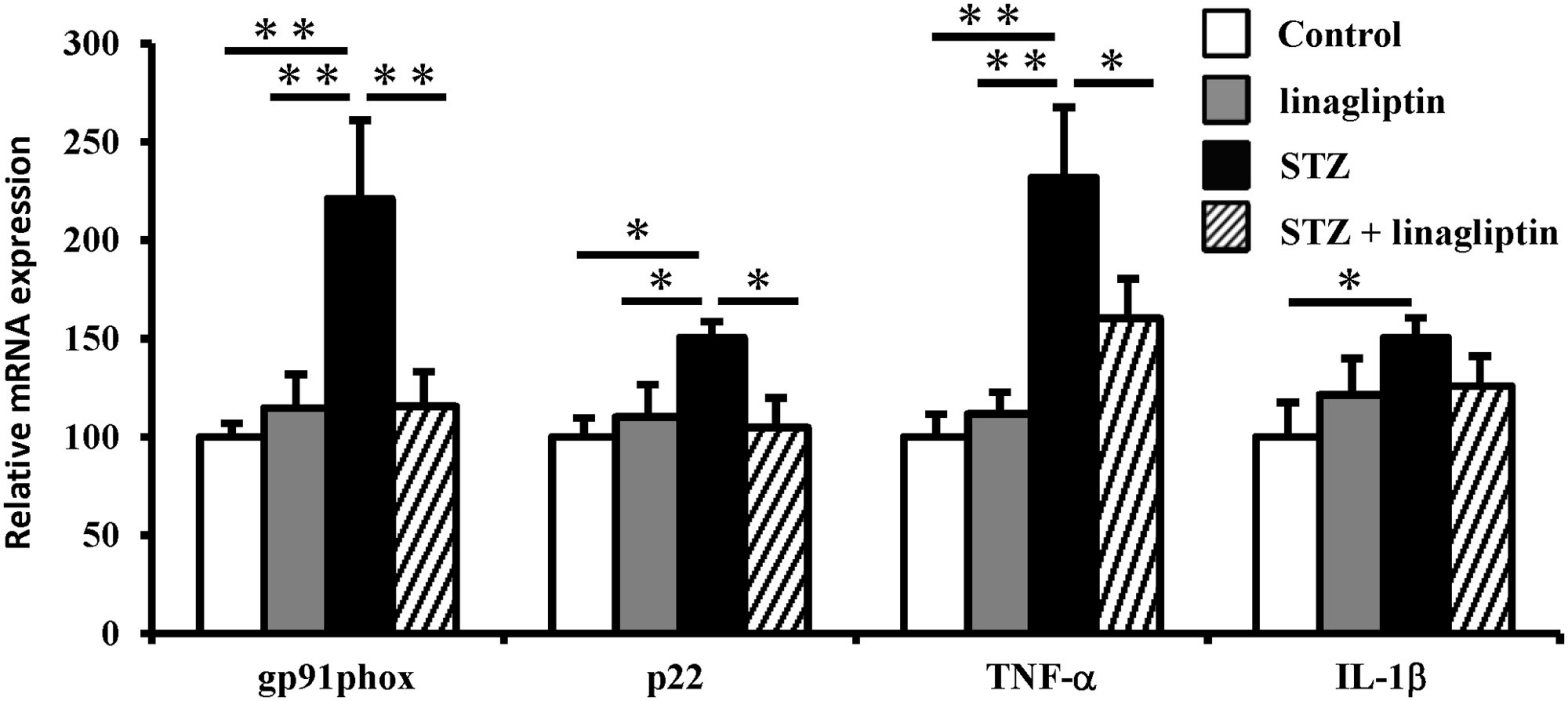
Effect of linagliptin on the mRNA expression of NAD(P)H oxidase components and proinflammatory cytokines in the brain. *gp91phox*, *p22*, *TNF-α* and *IL-1β* mRNA levels were measured by real-time RT-PCR and normalised against the *β-actin* levels. Results are expressed as means ± SE. (n = 8). *P < 0.05, **P < 0.01.

Microglia continuously monitor the brain environment[22,23]. In response to brain injury or immunological stimuli, microglia are readily activated, leading to proliferation and morphological changes[24]. Because we have recently reported that microglia are activated in diabetes-related cognitive impairment[6], we examined their cell number and body area using antibodies against Iba1, which is a protein restricted to microglia/macrophages[25] that is upregulated in activated microglia[26]. In STZ-induced diabetic mice, the number and body area of Iba1-positive microglia were significantly increased in the cerebral cortex compared with the control mice; this increase was reduced by linagliptin (Fig 4). Because activated microglia release superoxide radicals via NAD(P)H oxidase[27], we examined the expression of NAD(P)H oxidase components in the diabetic mice’s brain. As shown in Fig 5, compared with the control, the mRNA levels of the NAD(P)H oxidase components, *gp91phox* and *p22 phox*, were markedly increased in the STZ-induced diabetic mice, which was reduced by linagliptin. These results suggest that linagliptin improved cognitive impairment by suppressing NAD(P)H oxidase-mediated oxidative stress. Additionally, activated microglia produce the proinflammatory cytokines, tumour necrosis factor-alpha (TNF-α) and interleukin-1beta (IL-1β), which are thought to play a major role in inducing neuronal injury[28]. Therefore, we examined proinflammatory cytokines in the diabetic mice’s brain. As shown in Fig 5, the mRNA levels of *TNF-α* and *IL-1β* were markedly increased in the STZ-induced diabetic mice. However, the increase in *TNF-α* was reduced by linagliptin. These data suggest that linagliptin improved cognitive impairment by suppressing the activity of microglia, which induce NAD(P)H oxidase-mediated oxidative stress and proinflammatory cytokines’ expression.

## Discussion

DPP-4 inhibitors are a new class of antihyperglycemic agents[7], which increase the level of active GLP-1 and GIP in the peripheral blood. We initially found that linagliptin improved cognitive impairment in diabetic mice independent of the glucose-lowering effect (Figs 1 and 2). A previous study has shown that a DPP-4 inhibitor improved cognitive dysfunction by increasing the GLP-1 concentration in the brain[10,11]. Recently, Nakaoku et al. also reported that linagliptin ameliorates high-fat induced cognitive decline in tauopathy model mice[29]. Several studies have demonstrated that GLP-1 alleviated learning and memory dysfunction[30] and that a GLP-1 analogue and GIP analogue prevented memory impairments[31,32]. Additionally, it has been reported that GLP-1 and GIP inhibit microglial activation[32,33] and that linagliptin increased plasma active GLP-1 and GIP levels[12,34]. In the present study, we showed that linagliptin decreased the cell number and body area of microglia in the diabetic brain. Taken together, the DPP-4 inhibitor, linagliptin, may improve cognitive dysfunction by inhibiting microglial activation via increased GLP-1 and GIP levels. While GLP-1 and GIP are major substrates of DPP-4, DPP-4 also cleaves other substrates including stromal cell-derived factor 1 (SDF1α), which may have improved cognitive dysfunction, at least in part, in the present study; however, further studies are necessary to confirm this hypothesis. In addition, recent study showed that linagliptin did not modulate cognitive decline of patients with type 2 diabetes mellitus over a median treatment duration of 2.5 years[35]. Thus, further long-term follow-up study is necessary.

Oxidative stress has been considered to be a factor contributing to the development of diabetes and its complications[4,36]. As we have reported that oxidative stress and inflammation by activated microglia lead to cognitive impairment in diabetic mice[6], we evaluated the effect of linagliptin treatment on oxidative stress and proinflammatory cytokines. Consistent with our previous report[6], oxidative stress and *TNF-α* expression were increased in the brain of diabetic mice, and this effect was ameliorated by linagliptin. We have previously shown that microglia release superoxide radicals via NAD(P)H oxidase[27]. In the current study, we showed that linagliptin decreased the high mRNA expression of *gp91phox* and *p22 phox*, which are major component of NAD(P)H oxidase, in diabetic mice. These data suggest that linagliptin may decrease oxidative stress in the brain by limiting the upregulation of NAD(P)H oxidase expression in microglia. Additionally, we and others have reported that DPP-4 inhibitors have direct antioxidative effects *in vivo* and *in vitro*[37–39]. DPP-4 inhibitors cannot pass through the blood-brain barrier (BBB) under normal conditions[7], however, diabetes causes BBB dysfunction[40]. Therefore, it is possible that linagliptin can pass through the BBB and directly alleviate oxidative stress in the brain of diabetic mice; however, further studies are necessary to examine permeability of BBB in STZ-induced diabetic mice.

In conclusion, we showed that the DPP-4 inhibitor, linagliptin, improved cognitive dysfunction, at least in part, by decreasing oxidative stress and inhibiting microglial activation in a diabetes model mouse. These findings provide new insight into the efficacy of linagliptin in diabetes-related dementia. The effectiveness of linagliptin should be further confirmed in human trials.

## Supporting information

S1 Table Nucleotide sequences of primers (Related to Fig 5). (DOCX)
