# Supplementary material for: The dipeptidyl peptidase-4 inhibitor, linagliptin, improves cognitive impairment in streptozotocin-induced diabetic mice by inhibiting oxidative stress and microglial activation
Source: PLoS One. 2020 Feb 7;15(2):e0228750. doi: 10.1371/journal.pone.0228750 (PMC7006898; doi:10.1371/journal.pone.0228750)
Supplement: S1 Table — (DOCX) [file pone.0228750.s001.docx]

**S1 Table. Nucleotide Sequences of Primers (Related to Figure 5)**

| **Gene** | **Forward primer (5′→3′)** | **Reverse primer (5′→3′)** |
| --- | --- | --- |
| *gp91phox* | ACTGCGGAGAGTTTGGAAGA | GGTGATGACCACCTTTTGCT |
| *p22 phox* | TGGCTACTGCTGGACGTTTCAC | CTCCAGCAGACAGATGAGCACAC |
| *TNF-α* | CTCCTGGCCAACGGCATGGAT | ATCGGCTGACGGTGTGGGTG |
| *IL-1β* | AAATACCTGTGGCCTTGGGC | CTTGGGATCCACACTCTCCAG |
| *β-Actin* | TGACAGGATGCAGAAGGAGA | GCTGGAAGGTGGACAGTGAG |

TNF-α, tumour necrosis factor-alpha; IL-1β, interleukin-1beta.
